# Hochuekkito attenuates anxiety-like behavior associated with pulmonary inflammation induced by intratracheal lipopolysaccharides in mice

**DOI:** 10.3389/fphar.2026.1774957

**Published:** 2026-04-30

**Authors:** Yasuhisa Izushi, Soichiro Ushio, Teppei Ueda, Yuichi Tasaka, Ikuko Miyazaki, Masato Asanuma, Yoshihisa Kitamura

**Affiliations:** 1 Department of Pharmacotherapy, Graduate School of Pharmacy, Shujitsu University, Okayama, Japan; 2 Department of Pharmaceutical Sciences for Health Crisis Management, Faculty of Pharmaceutical Sciences, Fukuoka University, Fukuoka, Japan; 3 Laboratory of Clinical Pharmacy, School of Pharmacy, Shujitsu University, Okayama, Japan; 4 Department of Medical Neurobiology, Graduate School of Medicine, Dentistry and Pharmaceutical Sciences, Okayama University, Okayama, Japan

**Keywords:** anxiety, hochuekkito, inflammation, interleukin-6, lipopolysaccharide, lung, traditional Japanese herbal medicine

## Abstract

**Introduction:**

We have previously demonstrated that intratracheal lipopolysaccharide (LPS) injection induces significant pulmonary inflammation accompanied by hippocampal microglial activation, indicative of neuroinflammation. Hochuekkito (HET) is a traditional Japanese herbal medicine used to treat various conditions, including mental disorders and physical weakness. We have previously reported that HET ameliorates anxiety-like behaviors induced by intraperitoneal LPS injections in mice. However, its anxiolytic effects on anxiety-like behaviors due to pulmonary inflammation remain poorly understood. Therefore, in the present study, we aimed to investigate the effects of HET on anxiety-like behaviors induced by intratracheal LPS injection in mice.

**Methods:**

Mice received HET (1.0 g/kg) once daily for 2 weeks through oral gavage prior to LPS treatment. The light-dark box test was conducted 24 h following LPS injection to assess anxiety-like behaviors. Diazepam, a clinically used anxiolytic, served as a positive control. The lung wet-to-dry weight ratio was determined, and the concentrations of interleukin-6 (IL-6) in the lungs and serum were assessed.

**Results:**

Repeated administration of HET prevented the development of anxiety-like behaviors and reduced serum IL-6 concentrations and hippocampal *Il6* mRNA expression levels in LPS-treated mice. Diazepam failed to exert significant effects in LPS-treated mice, whereas HET remained effective under inflammatory conditions. Moreover, LPS injections significantly increased the number of Iba-1-immunoreactive microglial cells in the CA1 region of the hippocampus, whereas this effect was suppressed by treatment with HET. In the bronchoalveolar lavage fluid (BALF), the LPS-induced increase in white blood cell count was significantly reduced by treatment with HET. Furthermore, HET alleviated LPS-induced pulmonary inflammation, as evidenced by decreased lung wet-to-dry weight ratios.

**Conclusion:**

This study suggests that inflammation induced by intratracheal LPS injection contributes to anxiety-like behaviors in mice and that IL-6 may play a key role in linking peripheral inflammation to neuroinflammatory responses. The anxiolytic effects of HET appear to be associated, at least in part, with the suppression of IL-6 elevation in both the periphery and the hippocampus, along with attenuation of microglial activation. Our findings suggest that HET may serve as a potential therapeutic agent for anxiety-like behaviors associated with pulmonary inflammation.

## 1 Introduction

Severe acute respiratory syndrome coronavirus 2 (SARS-CoV-2) remains a significant global health concern. Infection with SARS-CoV-2 is characterized by an exaggerated inflammatory response and infiltration of inflammatory mediators, including pro-inflammatory cytokines, into the lung tissue (Al-Jassas et al., 2022; Mehta et al., 2020; Pelaia et al., 2020). A primary concern regarding the SARS-CoV-2 infection is its sequelae, which include prolonged fatigue, anxiety, and depression. These observations suggest that development of pharmacological therapy to treat pulmonary inflammation can be effective to restore psychiatric symptoms with infections.

Intraperitoneal injections of lipopolysaccharide (LPS), a bacterial endotoxin that induces inflammation, increase the levels of interleukin-6 (IL-6) and tumor necrosis factor-alpha, triggering inflammatory responses in the serum and hippocampus of mice (Kitamura et al., 2019; Ushio et al., 2022). Previous studies have shown that neuroinflammatory responses in specific brain regions are associated with stress-related behavioral abnormalities. For instance, inflammatory responses in the medial prefrontal cortex (mPFC) have been linked to social avoidance and anxiety-related behavioral changes (Nie et al., 2018). In addition, we have previously shown that LPS administration enhances microglial activity in the hippocampus of mice (Kitamura et al., 2019). Behavioral studies in rodents have also demonstrated alterations in psychological functions, including anxiety-like behaviors and depressive states, following LPS injections (Izushi et al., 2025; Okawa et al., 2024; Ushio et al., 2022). In a previous study, we developed a mouse model of pulmonary inflammation induced by intratracheal injections of LPS and conducted a series of experiments (Izushi et al., 2025). We demonstrated that intratracheal LPS injection induces significant pulmonary inflammation accompanied by hippocampal microglial activation, indicative of neuroinflammation. Notably, mice exhibited anxiety-like behaviors following intratracheal LPS injection (Izushi et al., 2025), further highlighting the relationship between pulmonary inflammation and anxiety.

Hochuekkito (HET; Bu Zhong Yi Qi Tang in Chinese; Bo Jung Ik Gi Tang in Korean) is a traditional Kampo medicine widely prescribed for a range of conditions, including mental disorders and physical weakness (Koshikawa et al., 1998). We previously reported that HET ameliorates anxiety-like behaviors induced by intraperitoneal lipopolysaccharide (LPS) injections in mice, an effect associated with the suppression of LPS-induced increases in IL-6 levels (Ushio et al., 2022). These findings suggest that IL-6 contributes to inflammation-induced anxiety-like behaviors and that modulation of IL-6 signaling may underlie the anxiolytic effects of HET. However, whether HET alleviates anxiety-like behavior associated with pulmonary inflammation remains unclear. Therefore, in this study, we aimed to investigate the anxiolytic effects of HET on anxiety-like behaviors induced by intratracheal LPS injections in mice using the light-dark box test. We also evaluated the anti-inflammatory effects of HET on LPS-induced pulmonary inflammation to assess whether suppression of inflammation may contribute to its behavioral effects.

## 2 Materials and methods

### 2.1 Animals

This study was conducted according to the recommendations of the Guide for Animal Experiments of Shujitsu University, Okayama, Japan. The protocol was approved by the Animal Care and Use Committee of Shujitsu University (Approval Nos.: 055-1, 056-1, 057-1, 061-1). A total of 217 male ICR mice with an initial weight of 29–35 g were purchased from The Jackson Laboratory (Yokohama, Japan). Mice were housed in groups of six per cage under a constant light-dark cycle (lights on from 8 AM to 8 PM) and fed standard laboratory food and tap water in an air-conditioned room (23 °C ± 1 °C with approximately 60% relative humidity).

### 2.2 Drugs

**FIGURE 1:**
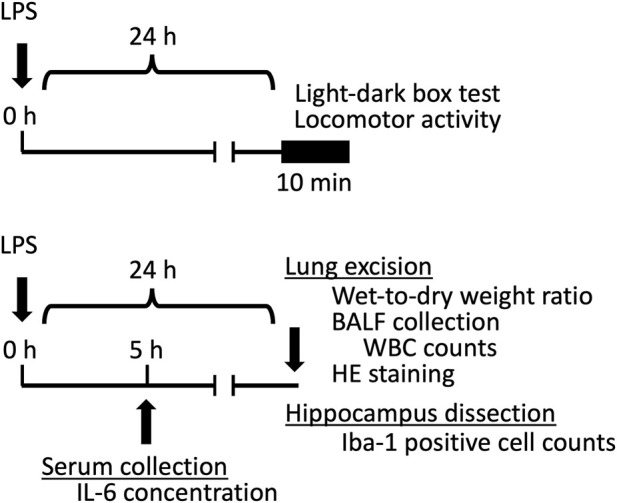
Schematic representation of the experimental design.

LPS (*Escherichia coli* O127:B8; Sigma-Aldrich, St. Louis, MO, United States) was dissolved in saline and administered intratracheally at a volume of 1 mL/kg body weight. For intratracheal instillation, a stainless-steel aerosol sprayer (KN-34700-1; Natsume Seisakusho Co., Ltd., Tokyo, Japan) (total length, 103 mm; straight portion, 80 mm; inner diameter, 0.3 mm; and outer diameter, 0.5 mm) attached to a gastight syringe (MS-GLL010; Ito Corporation, Tokyo, Japan) was used. The LPS dose (1 mg/kg) was selected based on our previous study (Izushi et al., 2025). HET (Lot No. 20160041010) was provided by Tsumura Co. (Tokyo, Japan). HET was used in the form of an extract powder composed of the following raw materials: 4.0 parts Japanese Pharmacopeia Astragalus root [*Astragalus membranaceus* Bunge, or *Astragalus mongholicus* Bunge (Leguminosae), radix], 4.0 parts JP Atractylodes lancea rhizome [*Atractylodes lancea* De Candolle, or *Atractylodes chinensis* Koidzumi (Asteraceae), rhizoma], 4.0 parts JP Ginseng [*Panax ginseng* C. A. Meyer (*Panax schinseng Nees*) (Araliaceae), radix], 3.0 parts JP Japanese angelica root [*Angelica acutiloba* Kitagawa, or *Angelica acutiloba* Kitagawa var. *Sugiyamae* Hikino (*Umbelliferae*), radix], 2.0 parts JP Bupleurum root [*Bupleurum falcatum* Linné (*Umbelliferae*), radix], 2.0 parts JP Jujube [*Ziziphus jujuba* Miller var. inermis Rehder (Rhamnaceae), fructus], 2.0 parts JP Citrus unshiu peel [*Citrus unshiu* Marcowicz, or *Citrus reticulata* Blanco (Rutaceae), pericarpium], 1.5 parts JP Glycyrrhiza [*Glycyrrhiza uralensis* Fischer, or *Glycyrrhiza glabra* Linné (Leguminosae), radix], 1.0 parts JP Cimicifuga rhizome [*Cimicifuga simplex* Turczaninow, *Cimicifuga dahurica* Maximowicz, *Cimicifuga foetida* Linné, or *Cimicifuga heracleifolia* Komarov (Ranunculaceae), rhizoma], and 0.5 parts JP ginger [*Zingiber officinale* Roscoe (Zingiberaceae), rhizoma]. Plants were identified by their external morphology and marker compounds, following the Japanese pharmacopeia and company standards. Extract quality was standardized based on good manufacturing practices, as defined by the Ministry of Health, Labor, and Welfare of Japan. The 10 herbs were boiled in purified water at 95 °C for 1 h. The liquid extract solution was filtered from the insoluble waste and reduced to a concentrate. The concentrate was then spray-dried to obtain the extract powder. The three-dimensional high-performance liquid chromatogram of the HET, provided by Tsumura & Co., is presented in Supplementary Figure S1. HET powder was dissolved in distilled water, and the doses were set based on previous reports (Cai and Yang, 2019; Tohda and Mingmalairak, 2013; Ushio et al., 2022). Dissolved HET was then administered via oral gavage (p.o.) at a volume of 10 mL/kg body weight. Glycyrrhizin (Tokyo Chemical Industry CO., LTD., Tokyo, Japan) was dissolved in distilled water and administered orally (p.o.) at a volume of 10 mL/kg body weight. Doses were set based on a previous report (Ushio et al., 2022). Diazepam (Wako Pure Chemical Industries, Ltd., Osaka, Japan) was suspended in 0.5% methylcellulose and administered via intraperitoneal (i.p.) injection at a volume of 10 mL/kg body weight. All experiments were conducted between 10 AM and 4 PM. Figure 1 depicts the experimental schedule, and Table 1 summarizes the treatment groups and corresponding treatments.

**TABLE 1:** Summary of the experimental groups and treatments.

Drugs	Pretreatment (days 1–14)	LPS administration
(A) HET experiments		
Control	Distilled water, p.o., once daily	Saline, i.t.
LPS	Distilled water, p.o., once daily	LPS 1 mg/kg, i.t.
HET	HET 1 g/kg, p.o., once daily	Saline, i.t.
LPS + HET	HET 1 g/kg, p.o., once daily	LPS 1 mg/kg, i.t.
(B) Glycyrrhizin		
Control	Distilled water, p.o., once daily	Saline, i.t.
LPS	Distilled water, p.o., once daily	LPS 1 mg/kg, i.t.
Glycyrrhizin	Glycyrrhizin 30 mg/kg, p.o., once daily	Saline, i.t.
LPS + Glycyrrhizin	Glycyrrhizin 30 mg/kg, p.o., once daily	LPS 1 mg/kg, i.t.

Drugs	Treatment	LPS administration
(C) Diazepam experiments		
Control	0.5% methylcellulose	Saline, i.t.
LPS	0.5% methylcellulose	LPS 1 mg/kg, i.t.
Diazepam	Diazepam 1 mg/kg, i.p.	Saline, i.t.
LPS + Diazepam	Diazepam 1 mg/kg, i.p.	LPS 1 mg/kg, i.t.

Mice were pretreated orally (p.o.) with distilled water or HET (1 g/kg, once daily) for 14 days, followed by intratracheal (i.t.) administration of saline or LPS (1 mg/kg). LPS, lipopolysaccharides; HET, hochuekkito.

Mice were pretreated orally (p.o.) with distilled water or glycyrrhizin (30 mg/kg, once daily) for 14 days, followed by intratracheal (i.t.) administration of saline or LPS (1 mg/kg). LPS, lipopolysaccharides.

Mice received either 0.5% methylcellulose or diazepam (1 mg/kg, i.p.) before intratracheal (i.t.) administration of saline or LPS (1 mg/kg). LPS, lipopolysaccharides.

### 2.3 Light-dark box test

The light-dark box is an anxiogenic challenge used to evaluate the conflict between the inclination of an animal to explore new environments and its aversion to brightly lit areas (Ushio et al., 2022). Each group consisted of six mice injected with LPS (1 mg/kg) 1 day before the test. HET (1 g/kg) was administered once daily for 14 days until the day before the experiment. The light-dark box consisted of light (20 × 20 × 25 cm) and dark (black walls and floor, 20 × 20 × 25 cm) compartments separated by a partition with a single opening (5 × 8 cm) for passage from one compartment to the other. At the beginning of the experiment, each mouse was placed in the light box facing away from the dark box. Each mouse spent 10 min in the illuminated area. The behaviors of the mice were videotaped and subsequently evaluated by experimenters blinded to the conditions.

### 2.4 Locomotor activity

Locomotor activity was monitored for 10 min using an automated activity monitoring chamber (DAS-8; Neuroscience, Inc., Tokyo, Japan). The plastic chambers measured 28 (width) × 20 (length) × 13 cm (height). The assay was conducted the day after the final injection of HET or LPS. Different mice were used for locomotor activity and light-dark box tests.

### 2.5 Immunohistochemistry

Six mice from each group were transcardially perfused with ice-cold saline, followed by fixation with 4% paraformaldehyde and 0.35% glutaraldehyde in 0.1 M phosphate buffer (PB, pH 7.4) under anesthesia (0.75 mg/kg medetomidine, 4 mg/kg midazolam, and 5 mg/kg butorphanol) 24 h post-LPS injection. After perfusion, the brain was removed *en bloc*, post-fixed for 24 h with a fixative containing 4% paraformaldehyde in 0.1 M PB (pH 7.4), and cryoprotected in 15% sucrose in 0.1 M PB with sodium azide for approximately 24 h. Brains that had been snap-frozen with powdered dry ice were cut coronally on a cryostat into 20-µm-thick sections containing the dentate gyrus of the hippocampus. For staining, sections were collected in 10 mM phosphate-buffered saline (PBS) with 0.1% sodium azide. Standard free-floating immunohistochemistry was used to detect the ionized calcium-binding adapter molecule-1 (Iba-1)-immunopositive signals in the CA1 region of the hippocampus. An adjacent set of equally spaced brain sections containing the hippocampus was selected from all animals. Sections were soaked in 10 mM PBS containing 0.2% Triton X-100 (PBST) for 30 min at room temperature. After incubation with 1% normal goat serum in PBST for 30 min, the sections were incubated with a polyclonal rabbit anti-Iba-1 antibody (diluted 1:2,000 in PBST; Cat# 019-19741; FUJIFILM Wako Pure Chemical Corporation) for 18 h at 4 °C. The sections were then washed with PBST (5 min × 5) and incubated with an Alexa Fluor 488-conjugated goat anti-rabbit IgG antibody (diluted 1:1,000 in PBST; Cat# A11034; Invitrogen, Carlsbad, CA, United States) for 2 h at room temperature. All slides were analyzed under a fluorescence microscope (Keyence Corporation, BZ-X800, Osaka, Japan) using a mercury lamp and a 470–490 nm wavelength filter to excite the Alexa Fluor 488 dye. The light emitted from the Alexa Fluor 488 dye was collected through a 515–550 nm bandpass filter, and stained cells were photographed at 20 × magnification. The number of Iba-1-positive cells on each side of the CA1 region of the hippocampus (−1.58 to −2.30 mm from the bregma) was counted in three arbitrary hippocampal sections per mouse. Each set contained sections of 20 µm thickness, covering the entire anteroposterior extent of the hippocampus. The number of Iba-1-positive cells in the CA1 region was counted by an investigator blinded to the injection groups.

### 2.6 Lung wet-to-dry weight ratio

Lungs were collected from six mice in each group under anesthesia 24 h following LPS injection. Mice were anesthetized with medetomidine (0.75 mg/kg), midazolam (4 mg/kg), and butorphanol (5 mg/kg). The lung wet-to-dry weight ratio was measured in six mice as an indicator of pulmonary edema. The tissue was dried in a constant temperature drying oven (DS601, Yamato Scientific Co., Ltd., Tokyo, Japan) at 60 °C for 72 h, and the dry weight was measured (Yang et al., 2025).

### 2.7 Bronchoalveolar lavage fluid (BALF) collection

Six mice from each group were injected with LPS 1 day before locomotor activity measurement. Mice were anesthetized with medetomidine (0.75 mg/kg), midazolam (4 mg/kg), and butorphanol (5 mg/kg). BALF was collected by washing the airways with 1.0 mL of ice-cold PBS and centrifuged at 400 × *g* for 10 min at 4 °C. BALF cells were resuspended in 0.5 mL Türk’s solution (Sigma-Aldrich, St. Louis, MO, United States). Total white blood cell (WBC) count was then determined using a hemocytometer.

### 2.8 Hematoxylin-eosin (HE) staining

Left lungs were collected from five mice per group after perfusion and fixation under anesthesia, as described above. Formaldehyde-fixed tissues were gently washed with 0.1 M PB overnight. The samples were then placed in 70% ethanol for 20 min (45 °C), dehydrated in 100% ethanol for 100 min (45 °C), and subsequently immersed in ethanol/xylene A and B for 40 min each (45 °C). Thereafter, they were soaked in xylene for 40 min (45 °C). Subsequently, the pieces were placed in paraffin tanks for 80 min (63 °C). From a paraffin-embedded lung tissue block, 3-µm-thick sections were cut and mounted on glass slides (Matsunami, Osaka, Japan). Thereafter, sections were deparaffinized and stained with Mayer’s hematoxylin solution (FUJIFILM Wako Pure Chemical Corporation) for 5 min, then rinsed with distilled water. The sections were stained with 0.5% eosin Y in ethanol (FUJIFILM Wako Pure Chemical Corporation, Tokyo, Japan) for 5 min, followed by dehydration through a graded ethanol series using Tissue Dehydration Solution A (FUJIFILM Wako Pure Chemical Corporation, Osaka, Japan). To evaluate morphological changes associated with pulmonary inflammation, 10 random lung fields per HE-stained section were captured at 20× magnification using a light microscope (BZ-X800; Keyence Corporation, Osaka, Japan). Image processing was performed using the BZ-X800 analysis application (1.1.30.19; Keyence Corporation), and the alveolar interstitial and alveolar air space areas were measured in µm^2^. Bronchial and pulmonary vascular structures were excluded from the analysis. The ratio of alveolar interstitial/alveolar air space was calculated to evaluate the thickness of the alveolar interstitium due to inflammatory cell infiltration, as previously described (Mouded et al., 2009).

### 2.9 Measurement of serum and lung interleukin (IL)-6 concentrations

Serum and lung IL-6 concentrations were measured to elucidate the effect of HET on mice receiving LPS injections. Blood samples were collected from six mice through decapitation 5 h after the final injection of LPS and HET. The middle lobe of the right lung was collected via decapitation 24 h after the final injection of LPS and HET. Blood was allowed to coagulate for 1 h at room temperature and 3 h at 4 °C. The coagulated blood was then centrifuged at 800 × g for 10 min at 4 °C to obtain serum. The middle lobe of the right lung was quickly removed and homogenized in ice-cold RIPA buffer (Santa Cruz Biotechnology, Dallas, TX, United States). As with the whole lung lysates, the homogenates were centrifuged at 20,000 × g for 30 min at 4 °C, and the supernatants were stored at −80 °C. IL-6 concentrations were determined via enzyme-linked immunosorbent assay using mouse IL-6 antibody pairs (R&D Systems, Minneapolis, MN, United States), according to the manufacturer’s instructions. All standards and samples were run in duplicate, and the values were interpolated using the obtained standard curves.

### 2.10 Measurement of *Il6* mRNA expression by real-time quantitative polymerase chain reaction

Hippocampal tissues were collected from individual mice by decapitation 5 h after LPS injection, and the dissected hippocampi were used to measure *Il6* mRNA expression. Total RNA was isolated from each hippocampus using the Maxwell® RSC Simply RNA Tissue Kit (Promega, Madison, WI, United States). Reverse transcription was performed using 0.25 μg of total RNA per sample with ReverTra Ace® qPCR RT Master Mix with gDNA Remover (TOYOBO, Osaka, Japan). Real-time PCR was conducted using THUNDERBIRD® Next SYBR™ qPCR Mix (TOYOBO, Tokyo, Japan) on a QuantStudio® 3 Real-Time PCR System (Thermo Fisher Scientific, Waltham, MA, United States). Primer sequences, target genes, and related information are provided in Table 2. The PCR protocol consisted of an initial denaturation at 95 °C for 20 s, followed by 40 cycles of denaturation at 95 °C for 3 s and annealing/extension at 60 °C for 30 s. Reaction specificity was confirmed by melting-curve analysis. Relative Il6 mRNA expression was normalized to *Gapdh* and calculated using the comparative cycle threshold (ΔΔCt) method (Livak and Schmittgen, 2001).

**TABLE 2:** Oligonucleotide sequences of the primer sets used for reverse transcriptase–polymerase chain reaction.

Gene symbol	Accession number	Forward (5ʹ to 3ʹ)	Reverse (5ʹ to 3ʹ)	Amplicon (bp)	Position (5ʹ to 3ʹ)
*Il6*	NM_031168.2	cca​ctt​cac​aag​tcg​gag​gct​ta	gca​agt​gca​tca​tcg​ttg​ttc​ata​c	112	218–329
*Gapdh*	NM_001411840.1	tgc​ccc​cat​gtt​tgt​gat​g	ggc​atg​gac​tgt​ggt​cat​ga	160	405–564

### 2.11 Statistical analysis

Data are expressed as the mean ± standard error of the mean (SEM). Data were analyzed using the Student’s t-test or one-way analysis of variance, followed by Tukey’s test (Excel-Tokei ver. 7.0; Esumi Co. Ltd., Tokyo, Japan) to determine the differences between the groups. Statistical significance was set at p < 0.05.

## 3 Results

### 3.1 Effects of HET on LPS-induced anxiety-like behavior in the light-dark box test and locomotor activity in mice

**FIGURE 2:**
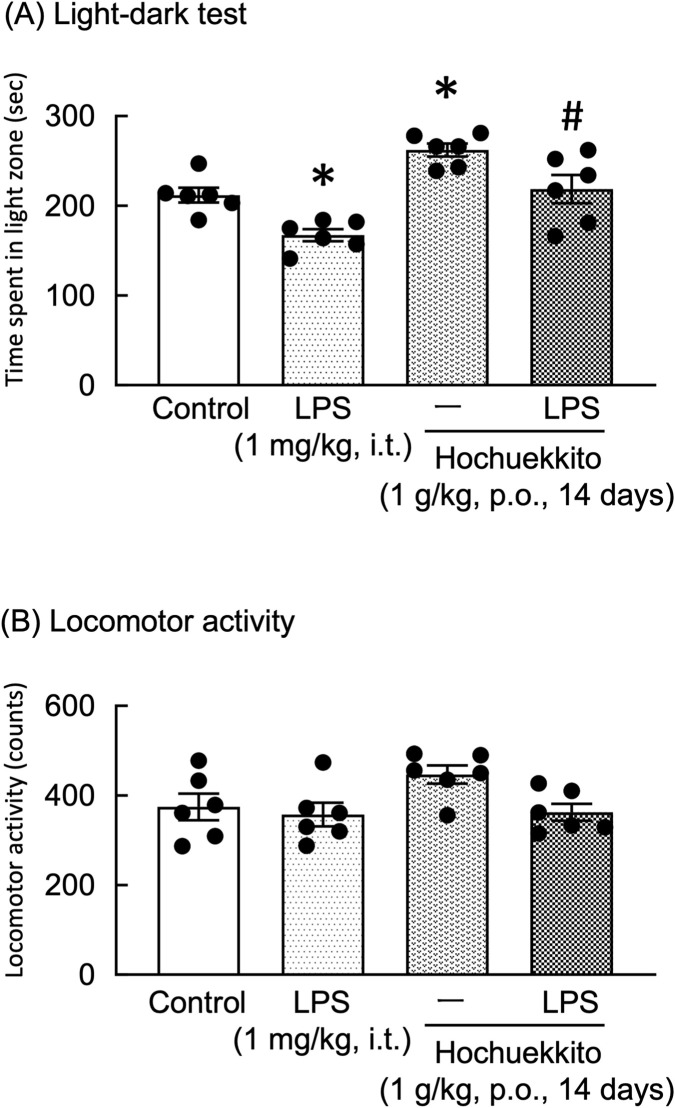
Effects of HET on **(A)** the time spent in the light zone in the light-dark test and **(B)** locomotor activity in mice following intratracheal LPS injection. HET (1 g/kg, p.o.) was administered once daily for 14 consecutive days prior to behavioral testing. LPS (1 mg/kg, i.t.) was administered 24 h prior to testing. Data are presented as the mean ± SEM; n = 6 per group. Statistical analysis was performed using one-way ANOVA, followed by Tukey’s *post hoc* test. *p < 0.05 (vs. control), #p < 0.05 (vs. LPS). LPS, lipopolysaccharides; HET, Hochuekkito.

In the light-dark test, the light-dark box represents an anxiogenic challenge that tests the conflict between the desire to explore new environments and the aversion to brightly lit zones. Our results indicated that treatment with LPS significantly reduced the time mice spent in the light zone. In contrast, HET significantly increased the time the mice spent in the light zone in both control and LPS-treated mice [F(3,20) = 14.61, p < 0.01; Figure 2A]. Locomotor activity in these treatment groups exhibited no significant changes [F(3,20) = 2.94, p = 0.06; Figure 2B].

### 3.2 Effects of glycyrrhizin on LPS-induced anxiety-like behavior in the light-dark box test

Glycyrrhizin, a major component of HET, significantly increased the time mice spent in the light zone in both control and LPS-treated mice [F(3,20) = 4.19, p < 0.05; Table 3].

**TABLE 3:** Effects of glycyrrhizin on the time spent in the light zone in the light-dark box test in mice following intratracheal LPS injection in mice.

Drugs	Time spent in light zone (s)
Control	223.8 ± 10.5
LPS	173.2 ± 10.7 *
Glycyrrhizin	215.3 ± 13.1
LPS + Glycyrrhizin	218.2 ± 10.9^#^

Glycyrrhizin (30 mg/kg, p.o.) was administered once daily for 14 consecutive days prior to behavioral testing. LPS (1 mg/kg, i.t.) was administered 24 h prior to testing. Data are presented as the mean ± SEM; n = 6 per group. Statistical analysis was performed using one-way ANOVA, followed by Tukey’s *post hoc* test. *p < 0.05 (vs. control), #p < 0.05 (vs. LPS). LPS, lipopolysaccharides.

### 3.3 Effects of diazepam on LPS-induced anxiety-like behavior in the light-dark box test

Diazepam, a benzodiazepine receptor agonist, was used as a positive control. Our results indicated that diazepam significantly increased the time the mice spent in the light zone. However, it did not increase the time spent in the light zone by LPS-treated mice [F(3,20) = 13.11, p < 0.01; Table 4].

**TABLE 4:** Effects of diazepam on the time spent in the light zone in the light-dark box test in mice following intratracheal LPS injection in mice.

Drugs	Time spent in light zone (s)
Control	195.8 ± 4.2
LPS	132.7 ± 15.4 *
Diazepam	258.8 ± 19.7 *
LPS + Diazepam	146.0 ± 19.0

Diazepam (1 mg/kg, i.p.) was administered 30 min prior to behavioral testing. LPS (1 mg/kg, i.t.) was administered 24 h prior to testing. Data are presented as the mean ± SEM (n = 6 per group). Statistical analysis was performed using one-way ANOVA, followed by Tukey’s *post hoc* test. *p < 0.05 (vs. control). LPS, lipopolysaccharides.

### 3.4 Effects of HET on Iba-1-positive microglial cells in the hippocampal CA1 region of LPS-injected mice

**FIGURE 3:**
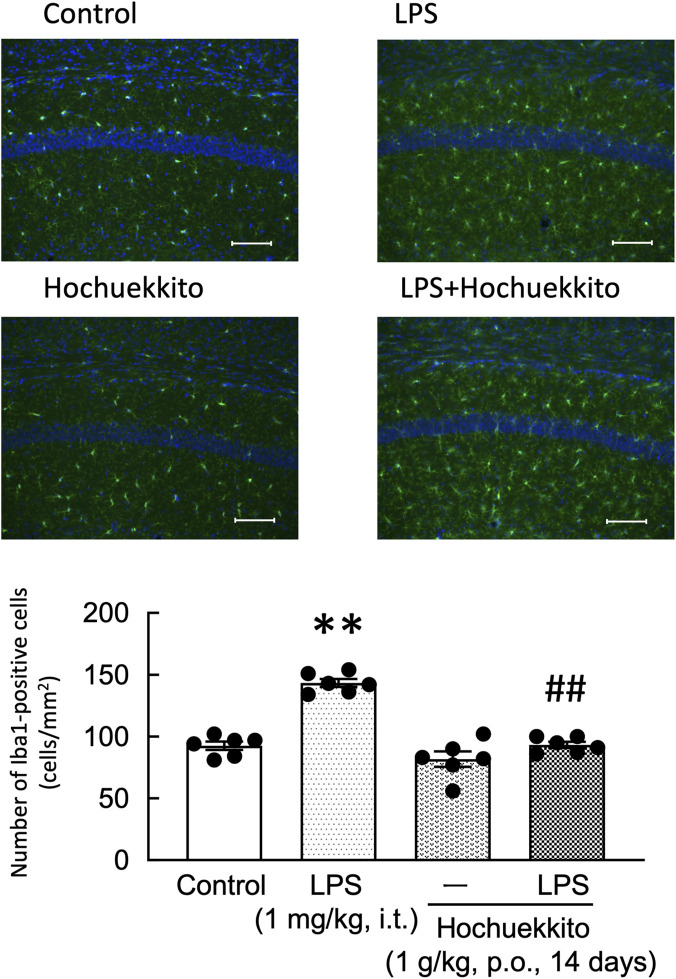
Effect of HET on the number of Iba-1-positive cells in the hippocampal CA1 region following intratracheal LPS injection. HET (1 g/kg, p.o.) was administered daily for 14 days. LPS (1 mg/kg, i.t.) was administered 24 h before tissue collection. Representative immunohistochemical images of Iba-1 staining in the CA1 region are depicted. Scale bar = 100 µm. Data are expressed as mean ± SEM; n = 6 per group. Statistical analysis was performed using one-way ANOVA, followed by Tukey’s *post hoc* test. **p < 0.01 (vs. control), ##p < 0.01 (vs. LPS). LPS, lipopolysaccharides; HET, Hochuekkito.

LPS injections significantly increased the number of Iba-1-immunoreactive microglial cells in the CA1 region of the hippocampus. In contrast, treatment with HET significantly attenuated this LPS-induced increase in Iba-1-positive microglial cells [F(3,20) = 45.56, p < 0.01; Figure 3]. Notably, similar effects were observed in the CA3 and dentate gyrus regions (data not shown).

### 3.5 Effects of HET on the lung wet-to-dry weight ratio in LPS-injected mice

**FIGURE 4:**
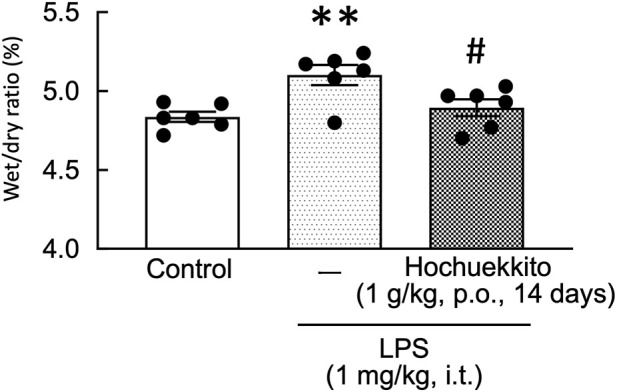
Effects of HET on the lung wet-to-dry weight ratio in mice following intratracheal LPS injection. HET (1 g/kg, p.o.) was administered once daily for 14 days. LPS (1 mg/kg, i.t.) was administered 24 h before lung tissue collection. Data are expressed as mean ± SEM; n = 6 per group. Statistical analysis was performed using one-way ANOVA, followed by Tukey’s *post hoc* test. **p < 0.01 (vs. control), #p < 0.05 (vs. LPS). LPS, lipopolysaccharides; HET, Hochuekkito.

The wet-to-dry weight ratio in the LPS group was significantly higher than that in the control group, whereas it was significantly decreased following treatment with HET [F(2,15) = 7.34, p < 0.01; Figure 4].

### 3.6 Effect of HET on the number of WBCs in the BALF of LPS-injected mice

**FIGURE 5:**
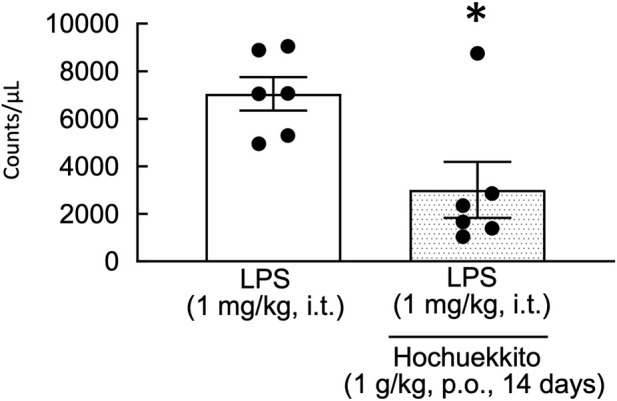
Effects of HET on WBCs in BALF in mice following intratracheal LPS injection. HET (1 g/kg, p.o.) was administered daily for 14 days. White blood cells in the BALF were counted 24 h following LPS (1 mg/kg, i.t.) administration. Data are presented as mean ± SEM; n = 6 per group. Statistical analysis was performed using an unpaired Student’s t-test. *p < 0.05 (vs. LPS). LPS, lipopolysaccharides; HET, Hochuekkito; WBC, white blood cell count; BALF, bronchoalveolar lavage fluid.

At 24 h post-LPS injection, HET significantly decreased the number of WBCs in the BALF of LPS-treated mice [t(10) = 2.94, p < 0.05; Figure 5].

### 3.7 Effect of HET on the pathomorphological changes in lung tissue of LPS-injected mice

**FIGURE 6:**
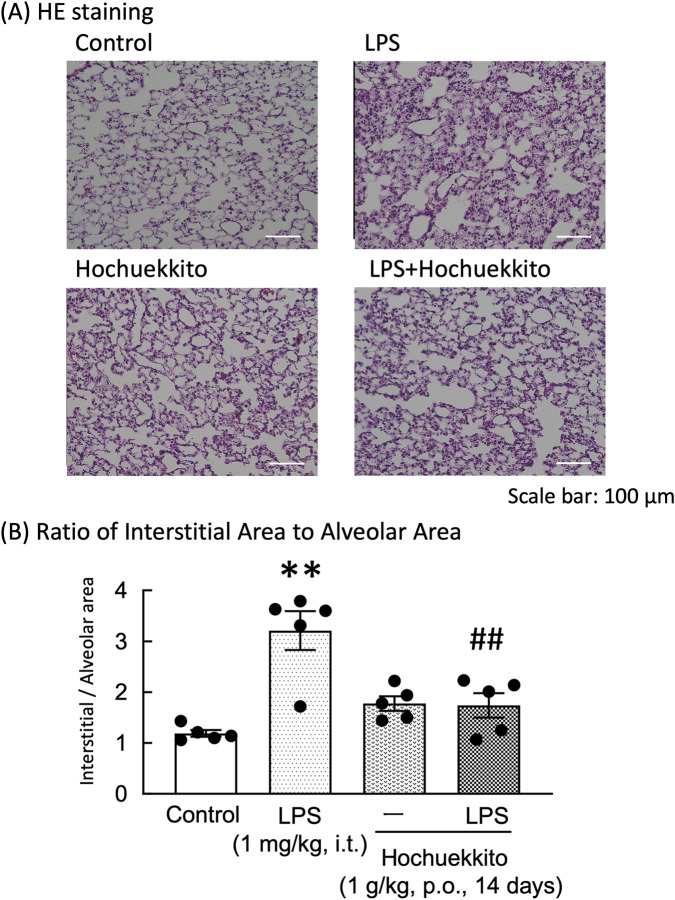
Effects of HET on lung histopathology following intratracheal LPS injection. **(A)** Representative HE-stained images of the left lung from the Control, LPS, Hochuekkito, and LPS + Hochuekkito groups. Scale bar = 100 µm. **(B)** Quantification of the ratio of interstitial area to alveolar area in each group. HET (1 g/kg, p.o.) was administered daily for 14 days. LPS (1 mg/kg, i.t.) was administered 24 h before lung tissue collection. Data are expressed as mean ± SEM; n = 5 per group. Statistical analysis was conducted using one-way ANOVA, followed by Tukey’s post hoc test. **p < 0.01 (vs. control), ##p < 0.01 (vs. LPS). LPS, lipopolysaccharides; HET, Hochuekkito.

Histological analysis with HE staining revealed that LPS injections caused notable pathological changes in lung tissue, including alveolar wall thickening and infiltration of inflammatory cells (Figure 6A). Moreover, the ratio of the interstitial to alveolar areas was significantly higher in the LPS than in the control groups. However, HET treatment significantly reduced this ratio in LPS-treated mice [F(3,16) = 13.15, p < 0.01; Figure 6B].

### 3.8 Effect of HET on serum and lung IL-6 concentrations in LPS-injected mice

**FIGURE 7:**
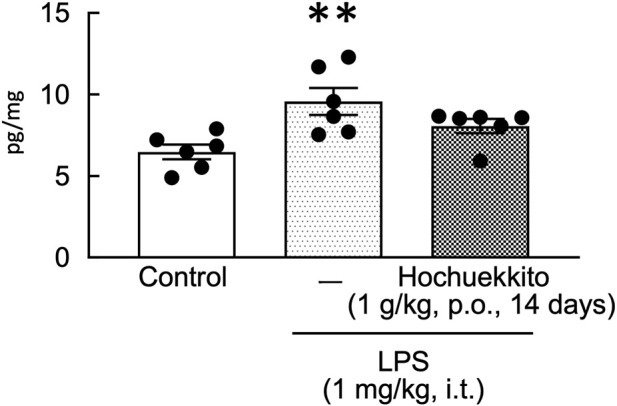
Effect of HET on IL-6 concentrations in the middle lobe of the right lung following intratracheal LPS injection. HET (1 g/kg, p.o.) was administered once daily for 14 days. IL-6 concentrations in the lung tissue were measured 24 h following LPS (1 mg/kg, i.t.) administration. Data are expressed as mean ± SEM; n = 6 per group. Statistical analysis was performed using one-way ANOVA, followed by Tukey’s *post hoc* test. **p < 0.01 (vs. control). LPS, lipopolysaccharides; HET, Hochuekkito; IL, interleukin.

In the control group, serum IL-6 concentrations were below the detection limit. Treatment with HET significantly attenuated the LPS-induced increase in serum IL-6 concentrations in LPS-injected mice at 5 h post-administration [t(10) = 3.17, p < 0.05; Table 5]. Similarly, IL-6 concentrations in the middle lobe of the right lung were significantly elevated following LPS injection, whereas this increase was suppressed by treatment with HET [F(2,15) = 6.72, p < 0.01; Figure 7].

**TABLE 5:** Effects of HET on serum IL-6 concentrations in mice 5 h following intratracheal injection of LPS in mice.

Drugs	IL-6 (pg/mL)
Control	ND
LPS	2578.7 ± 564.5
LPS + HET	800.8 ± 159.9 *

Hochuekkito (HET; 1 g/kg, p.o.) was administered once daily for 14 days. Serum IL-6 concentrations were measured 5 h following LPS (1 mg/kg, i.t.) injection. Data are expressed as mean ± SEM of the mean (n = 6 per group). Statistical analysis was performed using Student’s t-test. *p < 0.05 (vs. LPS). ND: not detectable. LPS, lipopolysaccharides; HET, hochuekkito.

### 3.9 Effect of HET on *Il6* mRNA expression in the hippocampus of LPS-injected mice

**FIGURE 8:**
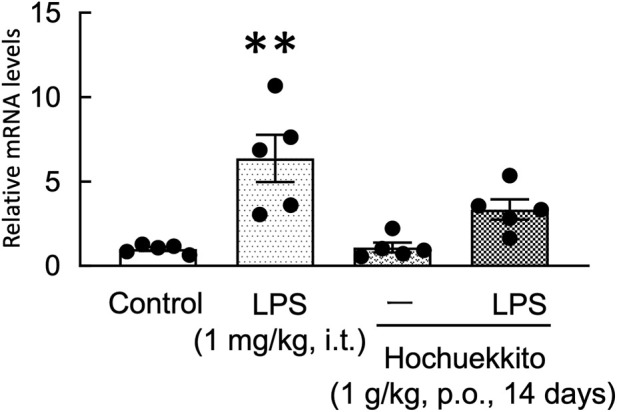
Effect of HET on hippocampal *Il6* mRNA expression following intratracheal LPS injection. HET (1 g/kg, p.o.) was administered once daily for 14 days. *Il6* mRNA levels in the hippocampus were measured 5 h after LPS (1 mg/kg, i.t.) administration. Data are expressed as mean ± SEM; n = 5 per group. Statistical analysis was performed using one-way ANOVA, followed by Tukey’s *post hoc* test. **p < 0.01 (vs. control). LPS, lipopolysaccharides; HET, Hochuekkito; *IL*, interleukin.

Hippocampal *Il6* mRNA expression was significantly increased 5 h after LPS administration. HET reduced *Il6* mRNA expression in the hippocampus of LPS-treated mice [F(3,16) = 10.52, p < 0.01; Figure 8].

## 4 Discussion

The anxiolytic effects of HET on anxiety-like behaviors induced by pulmonary inflammation remain unclear. We have previously demonstrated that intratracheal LPS injection induces anxiety-like behavior in mice, comparable to systemic inflammation (Izushi et al., 2025). In the present study, we further demonstrated that HET effectively alleviates the anxiety-like behavior resulting from intratracheal LPS-induced pulmonary inflammation. This finding is of particular importance, as it highlights a direct link between localized pulmonary inflammation and anxiety-like behavior. Overall, our findings suggest that HET may represent a promising therapeutic candidate for inflammation-related neuropsychiatric symptoms.

Neuroinflammation is characterized by the generation of pro-inflammatory cytokines and microglial hyperactivation (Leng and Edison, 2021). Consequently, the inhibition of microglial activation, which suppresses the release of pro-inflammatory mediators, is a crucial therapeutic approach for various diseases mediated by neuroinflammation (Wang et al., 2014). Our study demonstrated that intratracheal LPS injection substantially increased the number of Iba-1-positive cells, a marker of microglial activation, in the CA1 region of the hippocampus. Notably, this effect was significantly reduced by treatment with HET, suggesting that HET suppresses LPS-induced neuroinflammation.

In the present study, we selected the HET dose (1.0 g/kg) based on prior preclinical evidence. We previously demonstrated that HET at 1.0 g/kg produces remarkable anxiolytic-like effects in the light–dark test, whereas lower doses are ineffective (Ushio et al., 2022). Consistent with this, an independent study reported antidepressant-like effects of HET at the same dose in a learned-helplessness model (Tohda and Mingmalairak, 2013). Together, these findings support 1.0 g/kg as an effective and reproducible dose for evaluating the psychotropic effects of HET in rodent behavioral models. In addition to dose selection, the bioactive constituents of HET may contribute to the observed effects. A 3D-high-performance liquid chromatography chromatogram illustrating the chemical composition of HET is provided in Supplementary Figure S1, and several major components have been identified previously (Kao et al., 2001; Yamaya et al., 2007). For example, we previously reported that glycyrrhizin, a major constituent derived from *Glycyrrhiza* species and present in HET, ameliorates anxiety-like behavior induced by intraperitoneal LPS administration (Ushio et al., 2022). Here, we show that glycyrrhizin similarly attenuates anxiety-like behavior induced by intratracheal LPS administration. These results suggest that glycyrrhizin is one of the key constituents contributing to the anxiolytic-like effects of HET in this pulmonary inflammation model. In addition, *P. ginseng*, another constituent of HET, has been reported to attenuate stress-induced increases in serum IL-6 and to exert anxiolytic-like effects through both GABAA and serotonergic receptor systems (Ben-Azu et al., 2020). Collectively, these findings suggest that multiple herbal components of HET may act in concert to produce the anxiolytic-like effects observed in this study.

We utilized diazepam as a positive control in this study, as it is a representative anxiolytic drug that has long been used in clinical practice. However, it failed to exert a significant anxiolytic effect in mice subjected to intratracheal LPS administration, indicating that pulmonary inflammation attenuates its anxiolytic efficacy. We have previously reported similar findings in a systemic inflammation model, where diazepam paradoxically produced anxiogenic-like effects in the hole-board test following intraperitoneal LPS administration (Matsumoto et al., 2021). In our previous study, diazepam induced anxiety-like behavior under inflammatory conditions, possibly due to GABA_A_ receptor dysfunction associated with chloride plasticity mediated by the Na^+^-K^+^-2Cl^−^ cotransporter isoform 1 (NKCC1). Cumulatively, these findings suggest that LPS-induced neuroinflammation compromises the anxiolytic action of diazepam, explaining the reduced efficacy observed in the present study. A limitation of the present study is that anxiety-like behavior was assessed using only the light-dark box test. Although this paradigm is widely used and, together with our previous findings obtained using the hole-board test, supports the validity of the present behavioral interpretation, the inclusion of additional anxiety-related behavioral assays would further strengthen the construct validity of the anxiolytic-like effects of Hochuekkito. Future studies incorporating multiple behavioral paradigms, such as the hole-board test and/or elevated plus maze, are necessary to confirm the robustness of our findings.

Blocking IL-6 signaling with specific antibodies represents a promising therapeutic strategy for psychiatric disorders (Zhang et al., 2017; Zhou et al., 2017). Peripheral IL-6 expression plays a crucial role in the development of depression-like phenotypes following social defeat stress (Yang et al., 2015), and elevated serum IL-6 concentrations are strongly correlated with depressive and anxiety symptoms (Hodes et al., 2016). Furthermore, IL-6 has been reported to play a central role in pulmonary inflammation and injury, as previously reported in a study on SARS-CoV-2 (Zhu et al., 2025). It is also implicated in the pathogenesis of acute lung injury through the activation of the nuclear factor kappa B (NF-κB) signaling pathway (Zhang et al., 2025). In this study, we investigated whether intratracheal LPS injection induces anxiety-like behavior using the light-dark box test. We demonstrated that treatment with HET significantly attenuated LPS-induced anxiety-like behavior and markedly reduced serum IL-6 concentrations in mice exposed to intratracheal LPS injection. Collectively, these findings underscore the crucial role of IL-6 in the development of pulmonary inflammation, regardless of the underlying etiology. Elevated IL-6 concentrations are closely associated with increased vascular permeability, alveolar damage, and activation of inflammatory signaling pathways, such as NF-κB. In our study, HET administration effectively suppressed lung IL-6 production, alleviated pulmonary inflammation, and decreased pulmonary edema. These findings highlight IL-6 as a key mediator of lung injury and suggest that targeting IL-6 and its downstream inflammatory pathways may provide a promising therapeutic approach for inflammatory lung diseases.

Furthermore, in our study, histopathological analysis via HE staining revealed that intratracheal LPS injection induced pronounced inflammatory alterations in the lung tissue, characterized by alveolar wall thickening and robust inflammatory cell infiltration. Notably, pretreatment with HET markedly attenuated these pathological changes, suggesting a protective role against LPS-induced pulmonary inflammation. Mechanistically, the anti-inflammatory activity of HET appeared to involve dual modulation: suppression of serum IL-6 overexpression and attenuation of LPS-triggered significant elevation in IL-6 concentrations in the middle lobe of the right lung, both of which were effectively counteracted by HET. Moreover, HET significantly inhibited leukocyte recruitment and leukocyte accumulation in BALF, further corroborating its anti-inflammatory effects. Collectively, these data indicate that HET ameliorates LPS-driven lung dysfunction partly by suppressing IL-6-mediated pro-inflammatory signaling and limiting immune cell migration to pulmonary tissues. This mechanistic insight further highlights the therapeutic potential of HET in managing acute respiratory inflammatory disorders.

Importantly, accumulating evidence indicates that inflammatory signals originating in the periphery can propagate to the central nervous system. Central IL-6 signaling, particularly within limbic regions such as the hippocampus, plays a key role in regulating emotional behaviors, and inflammation-induced increases in hippocampal IL-6 have been linked to anxiety- and depression-like phenotypes (Deyama et al., 2017; Matsuura et al., 2023). Consistent with this framework, we found that intratracheal LPS administration significantly increased hippocampal Il6 mRNA expression 5 h after injection. This rapid induction suggests that pulmonary inflammation can influence central cytokine dynamics within a short time frame, potentially via humoral dissemination of inflammatory mediators and/or lung–brain neural communication pathways. Notably, HET attenuated the LPS-induced increase in hippocampal Il6 mRNA expression. Thus, suppression of hippocampal Il6 expression may be one mechanism by which HET ameliorates anxiety-like behavior. These findings also raise the possibility that HET exerts coordinated anti-inflammatory effects in both peripheral tissues and the central nervous system, thereby disrupting feed-forward signaling that links systemic inflammation to neuroinflammatory responses. Accordingly, the anxiolytic effects of HET may be mediated, at least in part, by attenuation of IL-6 induction following intratracheal LPS administration.

The present study has several limitations. First, phytochemical standardization or component-specific analyses of the HET extract was not performed. Therefore, the active constituents responsible for the observed anti-inflammatory and anxiolytic-like effects could not be identified. Future studies using analytical methods, such as liquid chromatography–mass spectrometry or high-performance liquid chromatography, will be needed to characterize the constituents of HET and to determine which compounds, or combinations of compounds, contribute to its pharmacological actions. Second, although our findings support the involvement of IL-6 signaling, the precise mechanisms by which HET exerts its anti-inflammatory and anxiolytic-like effects remain unclear. In addition to IL-6, other inflammatory mediators, including TNF-α, have also been reported to be modulated by HET in other experimental models and may contribute to its pharmacological effects (Vo et al., 2021). Further studies examining these established inflammatory targets are necessary to better define the mechanisms underlying the beneficial effects of HET in inflammation-associated anxiety-like behavior.

## 5 Conclusion

This study demonstrated that HET exerts significant protective effects against pulmonary inflammation-induced anxiety-like behavior in mice, partly by suppressing IL-6 production and attenuating inflammatory cell infiltration in the lung tissue. HET may offer a promising therapeutic strategy for managing neuropsychiatric symptoms associated with inflammatory lung diseases. Further studies are warranted to elucidate the molecular mechanisms underlying the anti-inflammatory and anxiolytic effects of HET and evaluate its clinical efficacy in patients with pulmonary inflammation-related neuropsychiatric disorders.

## Data Availability

The raw data supporting the conclusions of this article will be made available by the authors, without undue reservation.
