# Supplementary figures and images for: Hochuekkito attenuates anxiety-like behavior associated with pulmonary inflammation induced by intratracheal lipopolysaccharides in mice
Source: Front Pharmacol. 2026 Apr 30;17:1774957. doi: 10.3389/fphar.2026.1774957 (PMC13171584; doi:10.3389/fphar.2026.1774957)

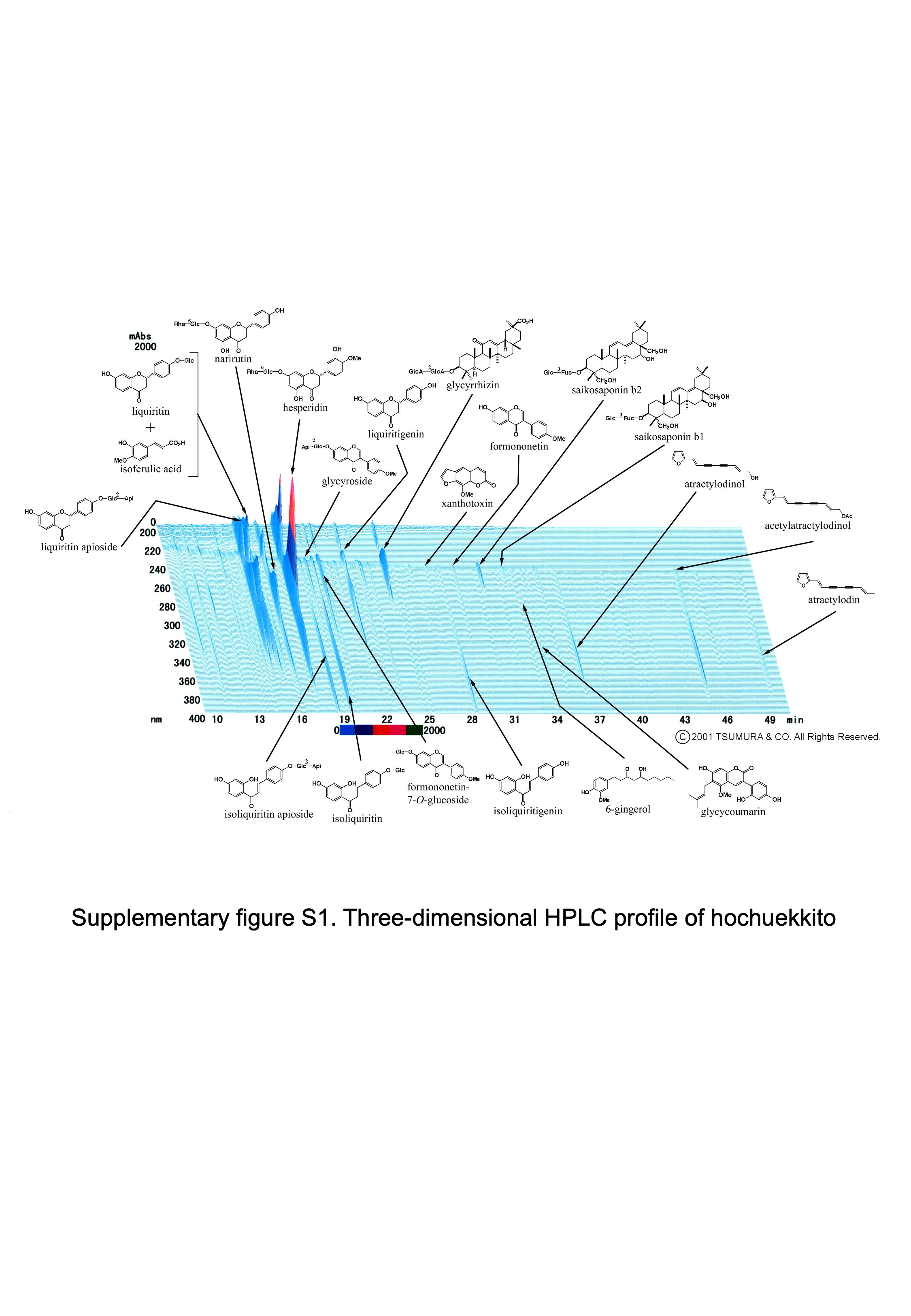

Supplement: Supplementary file 1 [file Image1.tiff]
